# Driftwood

**Published:** 1896-08

**Authors:** 


### DRIFTWOOD.

A Duluth, Minnesota, printer, has trained his two greyhound dogs to furnish the motor power for running his printing presses.

The smallest living horse in the world, of Orange County, Ohio, is the offspring of ordinary sized horses.

A number of claims have been found against the stockholders of the Ohio Veterinary College, at Cincinnati, by a referee appointed by the courts. The majority of the stockholders were found solvent. One of the stockholders proves to be a former dean of the school, but is noted as among those who are insolvent.

Miller's Hog-fever Cure Company is another corporation just organized in Iowa City, Iowa. Dr. John J. Miller, formerly a member of the U. S. V. M. A., but dropped from the rolls for failure to comply with its rules, is the moving spirit.

It is to be hoped that new editions of the Department of Agriculture books on *Diseases of the Horse* and on *Diseases of Cattle*, and *Cattle-feeding*, will not meet the fate of many of the first edition, in being offered for sale at a nominal figure by bookstores over the country.

There will be many changes in the college classes next year. Students are realizing the necessity of a higher education for entrance to practice in many of the States.

The horseshow in Washington Park, Chicago, on June 27th, was well attended. This is the first outdoor show ever given in the West, and will hereafter be an annual event, to replace old-time Derby Day. It is conducted under the auspices of the Northwestern Breeders' Association.

New York County has 328 registered veterinarians, 217 of the number by diplomas, the remainder by affidavit. Kings

County 112 registered veterinarians, 78 of whom have diplomas, the other 34 by affidavit.

Secretary Morton says that the adherence to civil-service rules in the Bureau of Animal Industry has improved the service, and the competency of the employés has been decidedly raised from the time the changes were made. This is a grand tribute to the merit-system of appointment.

